# Social Event Memory Test (SEMT): A Video-based Memory Test for Predicting Amyloid Positivity for Alzheimer’s Disease

**DOI:** 10.1038/s41598-018-28768-1

**Published:** 2018-07-10

**Authors:** Ko Woon Kim, Jong Doo Choi, Hyejoo Lee, Na Kyung Lee, Seongbeom Park, Juhee Chin, Byung Hwa Lee, Jiwon Shin, Yeshin Kim, Hyemin Jang, Jee Hyun Choi, Duk L. Na

**Affiliations:** 1 Department of Neurology, Samsung Medical Center, Sungkyunkwan University School of Medicine, Seoul, Korea; 2 Department of Neurology, Chonbuk National University Medical School & Hospital, Jeonju, Korea; 3 Convergence Research Center for Diagnosis, Treatment, and Care System of Dementia, Center for Neuroscience, Korea Institute of Science and Technology, Seoul, Republic of Korea; 4 Department of Health Sciences and Technology, SAIHST, Sungkyunkwan University, Seoul, Republic of Korea; 5 Sungkyunkwan University School of Medicine, Seoul, Korea; 6 Neuroscience Center, Samsung Medical Center, Seoul, Korea; 7 Department of Clinical Research Design & Evaluation, SAIHST, Sungkyunkwan University, Seoul, Korea

## Abstract

Recent improvements in neuroimaging and molecular markers of Alzheimer’s disease (AD) have aided diagnosis in the early stage of the disease, which greatly increases the chance for successful prevention and treatment. However, the expanding resources for AD diagnosis are unlikely to benefit all elderly due to economic burden. Here, we aimed to develop an inexpensive and sensitive method to detect early-stage AD. A scenario for real-world social event memory test (SEMT) was created and filmed in 360° video. Participants watched the 7-min video through head-mounted display (HMD) and then answered questionnaire about the video. We categorized the SEMT score into recall, recognition, and place-matching scores and compared them to scores on the Mini-Mental State Examination and Seoul Verbal Learning Test. Using the SEMT scores, we built a logistic regression model that discriminated between amyloid positivity and negativity of the participants, with a cross-validation AUC. Furthermore, a classifier was created using support vector machine, which produced 93.8–95.1% sensitivity in classifying individuals into four groups of normal, mild cognitive impairment with or without amyloid, and AD elderly. The high correlation between the SEMT score and amyloid positivity in individuals who experienced virtual social gathering through an HMD opens a new possibility for early diagnosis of AD.

## Introduction

Early intervention is crucial to improving the prognosis and reducing the morbidity associated with Alzheimer’s disease (AD). Therefore, early diagnosis is critical, and neuropsychological evaluation is one of the most efficient ways to screen individuals with cognitive impairment. Screening tests such as the Mini–Mental State Examination (MMSE) are short and easy to administer, but are not sensitive enough to detect amnestic type of mild cognitive impairment (aMCI)^1^. On the other hand, neuropsychological batteries have the advantage of analysing various domains of cognition in detail. However, the examination is time-consuming and has a high cost, leading to reduced motivation of the participants and failure to complete the test in some cases. Furthermore, standard neuropsychological tests are different from what people experience in ordinary daily life, which can produce participant discomfort during the test. In addition, most memory tests measure verbal and visual memory separately, although they are incorporated in reality. Thus, there is an urgent need to generate novel cognitive tests that are patient-friendly, less uncomfortable, and ecologically valid tests that can reflect cognitive function in daily life.

Unlike conventional cognitive tests, in our task called the Social Event Memory Test (SEMT), participants were asked to recall events after watching a short video clip that simulated a real-life social event: a birthday party scene where a host and six invitees casually converse about their personal lives. This video clip was designed based on previous studies reporting that episodic memory is more associated with increased amyloid burden than other cognitive domains^2,3^. Also, recent studies have shown that the capacity of associative memory, which involves linking two different pieces of information to each other (e.g., name and face, object and place), is significantly reduced in patients with amyloid depositions compared to those without amyloid depositions^4–7^. Therefore, the SEMT was designed to simultaneously evaluate verbal, visual, and associative memory.

Another feature that differentiates the SEMT from conventional tests is the use of a 360° video to create a memory test with the experience of a real-life social event. Recent fast-developing technology played an important role in creating a more realistic and interactive scenario that can be used to evaluate complex functional impairment under more natural conditions^8^. Providing a naturalistic and contextually rich “real-world” scenario to patients increases the ecological validity and reliability of neuropsychological assessments^9^. Ecological validity represents how close the settings of a study are to a real-world situation^10^. A higher level of ecological validity not only improves neuropsychological assessment, but also serves to identify the relationship between overall assessment results and cognitive impairment^11^. The use of an improved neuropsychological assessment to measure cognitive performance and dysfunction of the brain will be beneficial for diagnosing AD in its early stages. Therefore, we used a 360° camera and a head mount display (HMD) to increase the patients’ sense of reality during the examination.

The current study aimed to develop a user-friendly and real-life simulating neuropsychological test that is relatively short (playtime of 7.5 min) but sensitive enough to detect individuals at higher risk of developing dementia. We recruited three groups of participants: individuals with either subjective cognitive impairment (SCI)^3^, aMCI, or early stage AD. All participants underwent dementia work-ups that included detailed neuropsychological tests, blood tests, and magnetic resonance imaging (MRI). Participants also underwent [18 F] florbetaben (FBB) positron emission tomography (PET) scanning that is known to have high specificity and sensitivity for detecting amyloid deposition^12^. In this study, only amyloid negative SCI individuals^3^ and amyloid positive AD patients were recruited, whereas aMCI patients were divided into amyloid negative [aMCI(−)] and positive [aMCI(+)] groups following analysis of the amyloid scans. To validate the performance of the SEMT, it was first compared to standard tests such as the Korean version of the Mini-Mental State Examination (K-MMSE) and the Seoul Verbal Learning Test (SVLT)^13^. We then examined whether amyloid positive and negative groups can be differentiated using the SEMT and whether the SEMT can be used to classify patients into the following four diagnostic groups: SCI, aMCI(−), aMCI(+), and AD.

## Results

### Demographic and clinical characteristics

Table 1 summarizes the demographic and clinical features of the 52 participants who were divided into the following 3 groups: SCI (n = 13), aMCI (n = 25), and AD (n = 14). There were no significant differences among the groups in age, education, and gender. The SCI group received the highest MMSE scores, while the AD group received the lowest. *APOE* genotypes were performed for all participants, and the ratio of *APOE4* carriers differed between groups (ANOVA multiple comparison test, p = 0.0003) with the frequency of e4 allele being higher in the AD group (p = 0.0002) and the aMCI group (0.022), compared to that of the SCI group. Significant differences in memory (p < 0.0001), frontal/executive (p = 0.0005), and language (p = 0.0172) domain scores were noted among the SCI, aMCI, and AD groups. In the memory domain, the groups scored in the following order from highest to lowest: SCI, aMCI, and AD. In the frontal/executive domain, the SCI group received higher scores than the aMCI and AD groups.

**Table 1:** Demographic and clinical characteristics.

	Mean ± SD			P	Post-hoc Bonferroni		
	SCI	aMCI	AD		SCI vs aMCI	SCI vs AD	aMCI vs AD
**Age**, years	72.46 ± 3.86	75.48 ± 4.85	72.64 ± 6.79	0.1423			
**Education**, years	13.23 ± 3.85	12.92 ± 4.65	13.00 ± 3.80	0.9813			
**Gender**							
Female:Male	8:5	14:11	6:8	0.5956			
**APOE4 carrier (%)**	0/13 (0%)	11/25 (44%)	10/14 (71%)	0.0003	0.0220	0.0002	0.0790
**MMSE**	29.00 ± 1.08	25.72 ± 2.85	22.79 ± 2.39	<0.0001	0.0005	<0.0001	0.0065
**Domain score (max score)**							
Attention (17)	11 ± 2.80	9.24 ± 2.49	9.46 ± 2.22	0.1788			
Language (27)	24.62 ± 2.47	21.24 ± 3.91	22.31 ± 2.72	0.0043	0.0056	0.0360	1
Visuospatial (36)	33.77 ± 1.74	29.62 ± 6.01	29.62 ± 7.35	0.0349	0.0348	0.1746	1
Memory (150)	100.54 ± 12.27	47 ± 14.08	27.88 ± 8.92	<0.0001	<0.0001	<0.0001	0.0001
Frontal/Executive (70)	57.69 ± 8.41	44.60 ± 12.70	39.38 ± 11.55	0.0003	0.0037	0.0006	0.5262
Total (300)	227.62 ± 24.18	151.70 ± 27.21	128.65 ± 20.47	<0.0001	<0.0001	<0.0001	0.0203
**SVLT (max score)**							
Immediate (36)	25.23 ± 3.92	15.79 ± 3.88	11.15 ± 4.86	<0.0001	<0.0001	<0.0001	0.0067
Delayed (12)	8.69 ± 1.75	2.04 ± 1.90	0.31 ± 0.85	<0.0001	<0.0001	<0.0001	0.0090
**SEMT (max score)**							
Free recall (36)	17.15 ± 5.90	4.00 ± 4.79	0.36 ± 0.50	<0.0001	<0.0001	<0.0001	0.0062
Recognition (18)	11.54 ± 2.33	11.08 ± 1.63	10.57 ± 2.06	0.4377			
Place-matching (48)	29.31 ± 10.22	10.40 ± 7.34	4.71 ± 3.45	<0.0001	<0.0001	<0.0001	0.0784
Total (102)	58.00 ± 15.23	25.48 ± 12.23	15.86 ± 4.44	<0.0001	<0.0001	<0.0001	0.0087

AD: Alzheimer’s disease; aMCI: amnestic mild cognitive impairment; MMSE: Mini Mental State Examination; SCI: subjective cognitive impairment; SEMT: social event memory test; SVLT: Seoul Verbal Learning Test.

### Statistical parametric mapping of amyloid PET images

**Figure 1:**
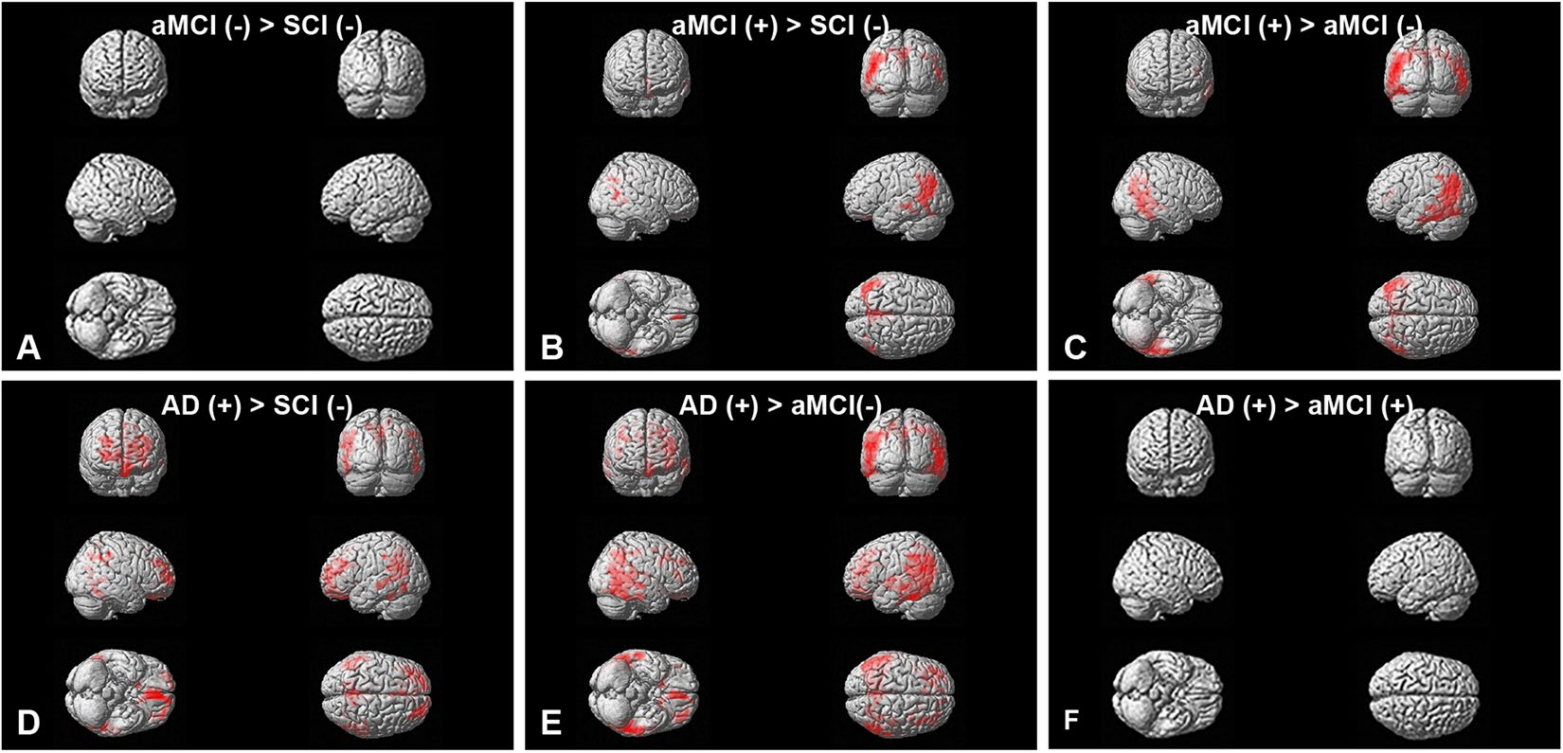
Amyloid PET image. Statistical parametric mapping of florbetaben (FBB) retention in SCI, aMCI(−), aMCI(+), and AD. AD and aMCI(+) groups demonstrated amyloid retention patterns in the bilateral frontal and temporoparietal cortices compared to aMCI(−) and SCI groups. (**A**) aMCI(−) > SCI; (**B**) aMCI(+) > SCI; (**C**) aMCI(+) > aMCI(−); (**D**) AD > SCI; (**E**) AD > aMCI(−); (**F**) AD > aMCI(+). All AD participants were amyloid positive, and all SCI participants were amyloid negative. The results are presented at a threshold of p < 0.05, FWE corrected. AD: Alzheimer’s disease; aMCI(−): amyloid negative amnestic mild cognitive impairment; aMCI(+): amyloid positive amnestic mild cognitive impairment; SCI: subjective cognitive impairment.

Statistical parametric mapping of florbetaben (FBB) retention was performed for the following groups: SCI, amyloid negative aMCI, amyloid positive aMCI, and AD. Cortical retention of FBB was significantly higher in the bilateral frontal and temporoparietal cortices of AD patients compared to amyloid negative SCI and aMCI patients (Fig. 1D,E). Additionally, cortical retention of FBB in the bilateral temporoparietal regions was significantly higher in the amyloid positive aMCI group compared to the amyloid negative SCI and aMCI groups (Fig. 1B,C). The results are presented at a threshold of p < 0.05, FWE corrected (Fig. 1).

### Comparison of SEMT scores among the three groups

The three groups (SCI, aMCI, and AD) received scores that significantly differed from each other for the following tests: SEMT free-recall (p < 0.0001), SEMT place-matching (p < 0.0001), MMSE (p < 0.0001), SVLT immediate recall (p < 0.0001), and SVLT delayed recall (p < 0.0001) (Table 1). In contrast, the three groups did not differ in SEMT recognition score (p = 0.4377) (Table 1). Within the subgroup of normal MMSE scores from 28 to 30, SEMT scores showed a significant relationship with amyloid positivity: amyloid (+) group showed lower scores in SEMT total (p = 0.008), SEMT free recall (p = 0.014), and SEMT place-matching (p = 0.008) than the amyloid (−) group (Supplementary Table S2). Furthermore, we performed a correlational analysis between the APOE genotype groups (non-carrier, heterozygous carrier, and homozygous carrier) and SEMT scores, resulting in a significant association between the APOE genotype and the SEMT place-matching score within the MCI group (r = −0.422, p = 0.040) (Supplementary Table S3).

### Correlation analysis of SEMT score with MMSE and SVLT scores

**Figure 2:**
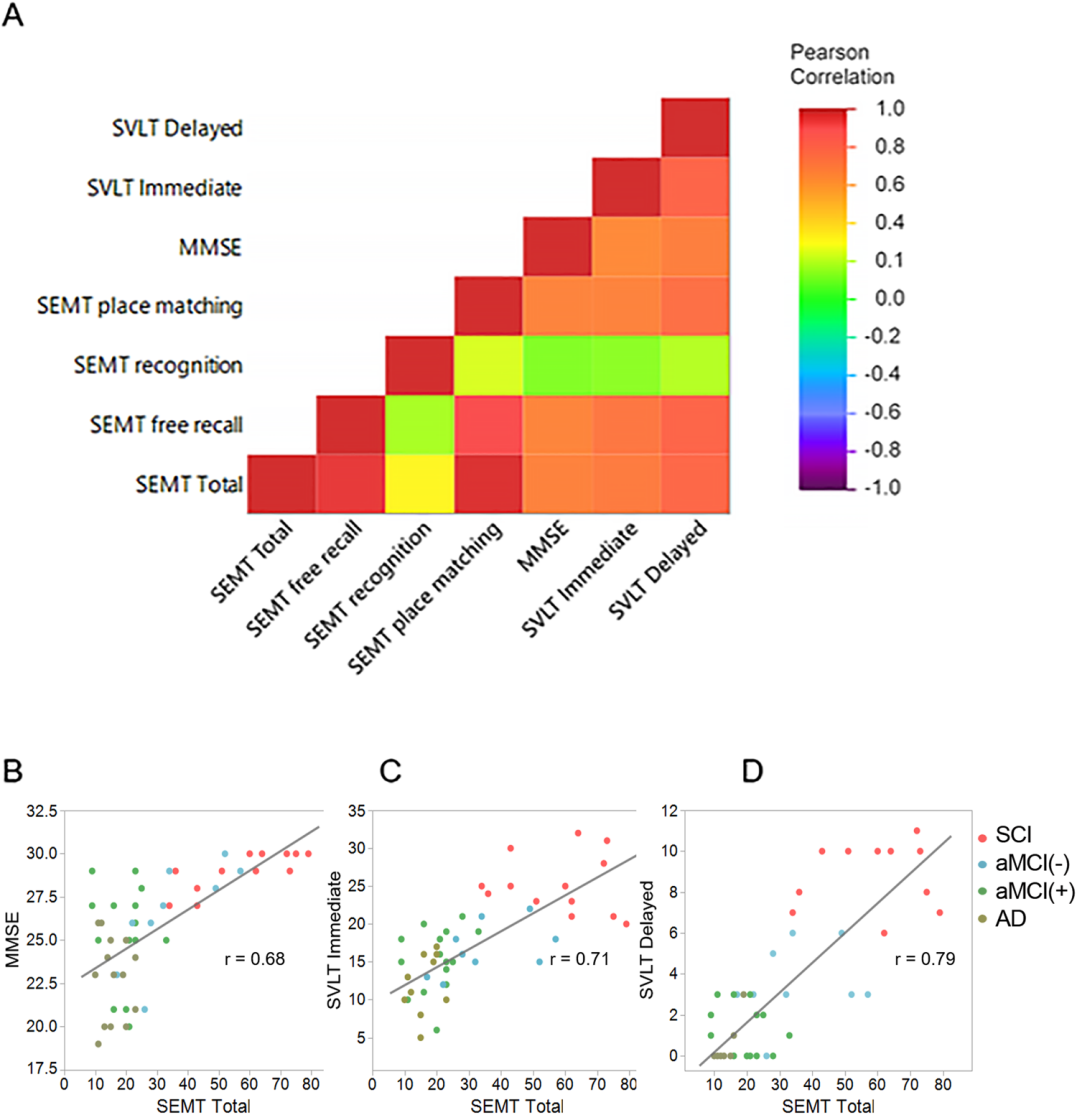
(**A**) Pairwise Pearson’s correlations between corresponding variables are plotted as a heat map matrix. Each cell in the heat map indicates the correlation coefficient using a rainbow coloured scale ranging from red (maximum correlation) to violet (minimum level of correlation). The strong, high positive correlation between SEMT, SVLT, and MMSE scores is clearly visible in the heat map. (**B**–**D**) Data set points were plotted as scatter plots: SEMT total versus (**B**) MMSE, (**C**) SVLT immediate, and (**D**) SVLT delayed. The fitted line is drawn in red. A strong correlation between SEMT total and MMSE, SVLT immediate, and SVLT delayed can be observed. AD: Alzheimer’s disease; aMCI(−): amyloid negative amnestic mild cognitive impairment; aMCI(+): amyloid positive amnestic mild cognitive impairment; MMSE: Mini Mental State Examination; SCI: subjective cognitive impairment; SEMT: social event memory test; SVLT: Seoul Verbal Learning Test.

The heat map of the correlation matrix shown in Fig. 2 indicates that the SEMT and SVLT scores significantly positively correlate with each other. Interestingly, however, the SEMT did not show a significant correlation with age or education. Pearson’s correlation analysis revealed significant relationships between SEMT score and other standard test scores. The p-values for the correlation analysis are shown in Supplementary Table S4.

### Correlation analysis of SEMT score with hippocampal volume

The SEMT score and hippocampal volume positively correlated with each other. Pearson’s correlation analyses revealed significant relationships between hippocampal volume and not only SEMT score but also other conventional cognitive test scores (MMSE and SVLT). The p-values for the correlation analysis are shown in Table 2.

**Table 2:** Pearson’s correlations between hippocampal volume and SEMT score.

	Total hippocampus	Right hippocampus	Left hippocampus
**SEMT Total**	0.6410	0.6276	0.6100
**SEMT free recall**	0.6929	0.6732	0.6643
**SEMT place-matching**	0.6303	0.5938	0.5938
All *p* < 0.0001 for Pearson correlation coefficient			
**SVLT immediate recall**	0.5631	0.5211	0.5657
**SVLT delayed recall**	0.5850	0.5486	0.5810
**SVLT recognition**	0.4122	0.3493	0.4445
All *p* < 0.01 for Pearson correlation coefficient			
**MMSE**	0.4330	0.3842	0.4494
All *p* < 0.05 for Pearson correlation coefficient			

MMSE: Mini Mental State Examination; SEMT: social event memory test; SVLT: Seoul Verbal Learning Test.

### Logistic regression model for prediction of amyloid positivity

Logistic regression analysis was conducted to determine whether FBB uptake is associated with SEMT score, which can eventually be used to predict the outcome of amyloid PET scans (Supplementary Table S5). The selected optimal regression model took the following equation form:

1 $$\begin{array}{ccc}\mathrm{ln}(\frac{p}{1-p}) & = & 10.51-o.31SEMT\,total-0.077{(SEMTtotal-30)}^{2}\\  &  & +\,0.33(SEMT\,total-30)\ast (SEMT\,free\,recall-5.9)\\  &  & -\,0.34{(SEMTfreerecall-5.9)}^{2}\end{array}$$

**Figure 3:**
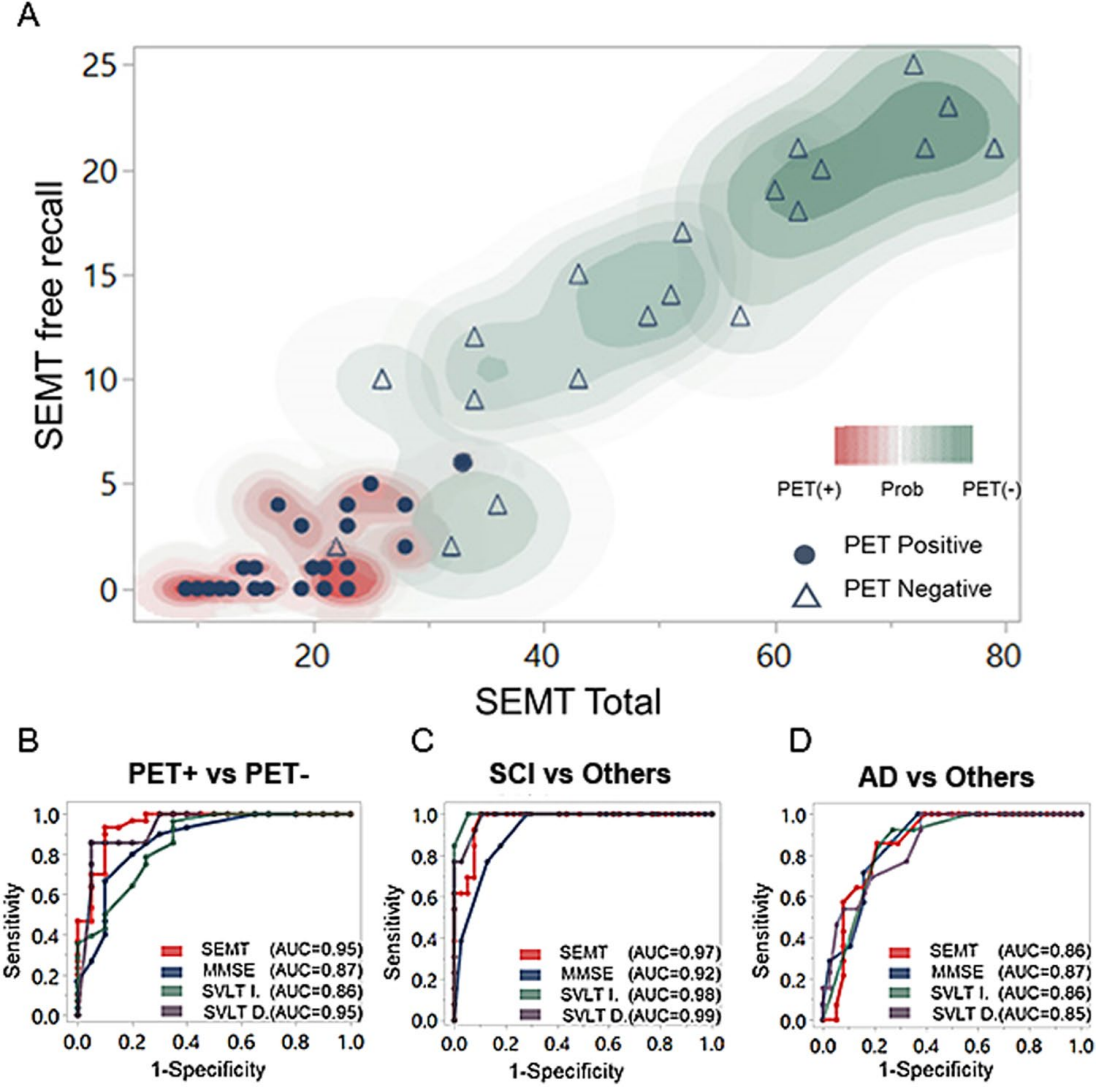
(**A**) Graphical representation of the regression model showing the probability of being amyloid positive or negative according to SEMT free recall and SEMT total scores. The contoured area represents the probability of amyloid PET (+) or amyloid PET (−) with SEMT free recall along the y-axis and SEMT total along the x-axis. (**B**) ROC curves plotted by sensitivity (y-axis) versus 1 - specificity (x-axis) to classify amyloid PET positive and negative cases, (**C**) SCI and other groups, and (**D**) AD and other groups. Although high AUC scores were obtained from the ROC analysis, this did not fully explain the data set. In classifying amyloid PET positive and negative, the R-squared was 0.6 for SEMT total, 0.65 for SVLT delayed, and less than 0.4 for MMSE and SVLT immediate. The R-squared values of all other analyses were below 0.5. AD: Alzheimer’s disease; aMCI(−): amyloid negative amnestic mild cognitive impairment; aMCI(+): amyloid positive amnestic mild cognitive impairment; MMSE: Mini Mental State Examination; PET: positron emission tomography; ROC: Receiver operator characteristic; SCI: subjective cognitive impairment; SEMT: social event memory test; SVLT D.: Seoul Verbal Learning Test delayed recall; SVLT I.: Seoul Verbal Learning Test immediate recall.

Equation (1) estimated the probability of obtaining amyloid PET positivity based on the SEMT total and SEMT free recall scores. Based on Chi-square analysis, the selected model fit fairly well (p < 0.0001). The R-squared of 0.78 indicated that 78% of the variation in response to variable Y can be explained by the predictors. The lack-of-fit test was used to observe whether the model fit well against the fit of the saturated model. The high p-value indicated that there was a selected model that fit better than the more complex model, and that adding more terms was not necessary. A graphical representation of the regression result is shown in Fig. 3A. Red contour areas illustrate the proportion of amyloid-positive patients predicted according to the regression model, while green contour areas represent the proportion of amyloid-negative patients. The plot was made using real data sets. From the contour plot, it was apparent that most of the participants were correctly identified, and only two points (individuals) were not predicted. Based on logistic regression analysis, a close association was observed between SEMT and amyloid PET outcomes, which indicated that the selected model provided significant coefficients to predict whether a patient had amyloid positive scans.

The receiver operator characteristic (ROC) curve was used to compare the performance of the SEMT to other standard neuropsychological tests such as the MMSE and SVLT. Figure 3B shows the ROC curves of different tests to classify participants as amyloid PET positive or amyloid PET negative. ROC curves of the SEMT and SVLT were above the ROC curves of other models, indicating that the performance of the SEMT and SVLT was better than that of other models.

In addition, we compared the SEMT with other standard neuropsychological tests (MMSE and SVLT) in terms of classifying participants into the SCI and other groups or into the AD and other groups. When classifying participants into the SCI and other groups, the ROC curve of the MMSE was below that of the other models, with the lowest area under the curve (AUC) of 92% (Fig. 3C). All of the models showed ROC curves with similar patterns and AUC values for classifying the AD group (Fig. 3D). All the R square statistics describing the proportion of total variance with respect to the ROC model were less than those of the logistic regression model.

### Classification of the four groups using support vector machine SEMT classifiers

**Figure 4:**
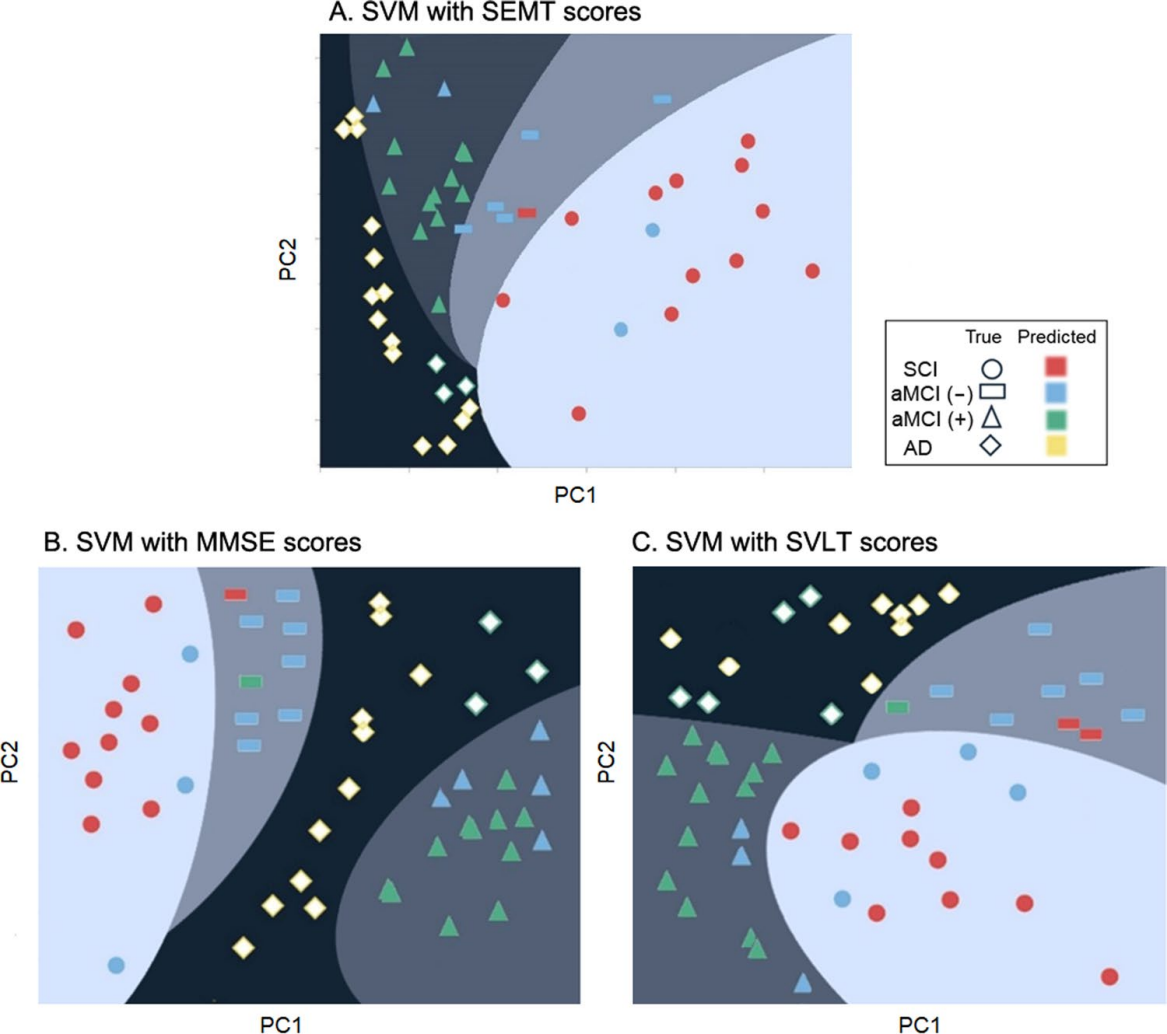
Plot of separated space using the SVM with different kernels by the reduced first two principal component scores from high dimensional space. (**A**) SVM result using SEMT scores (**B**) SVM result using MMSE scores (**C**) SVM result using SVLT scores. MMSE: Mini Mental State Examination; PC1: first principal component; PC2: second principal component; SEMT: social event memory test; SVLT: Seoul Verbal Learning Test; SVM: support vector machine.

Support vector machine (SVM) analysis yielded classifiers with high sensitivity and prediction accuracy for all four diagnostic groups: amyloid negative SCI, amyloid negative aMCI, amyloid positive aMCI, and amyloid positive AD. The performance of the SVM was evaluated by overall accuracy, sensitivity, specificity, positive predictive value (PPV), and negative predictive value (NPV). In our study, for instance, the sensitivity of SCI was measured by the proportion of positive predictions, which were SCI cases among actual positives. To explore the prediction accuracy of the SEMT in relation to diagnostic classification, SVM analysis was conducted by including the following variables: age, education, SEMT free recall score, SEMT recognition score, and SEMT place matching score. Two different types of kernel functions were used: linear and radial basis function (RBF). Two of the SVM analyses yielded classifiers with high prediction accuracy: 75% in the linear model and 85% in the RBF model. Compared to the linear kernel, the SVM with the RBF kernel performed better (Fig. 4). Performance results of the SVM with RBF are provided in Table 3. Interestingly, compared to other prediction measures, the SVM produced the highest sensitivity for all diagnostic groups (all sensitivities 93.8–95.1%). It is worthy to note that among the false prediction in the SCI group, 61.4% of patients were predicted as aMCI(−), and 38.6% of SCI were predicted as aMCI(+). For the false prediction in the aMCI(−) group, 43.6% were predicted as SCI, 55.7% were predicted as aMCI(+) and only 0.6% were predicted as AD. For the aMCI(+) group, 16.4% were misclassified as aMCI(−) and 83.6% were misclassified as AD. For the false prediction in AD patients, all patients were predicted as aMCI(+).

**Table 3:** Performance of SVM classifier models using SEMT, MMSE, and SVLT.

Modelling parameters	Subject group	Sensitivity (%)	Specificity (%)	PPV (%)	NPV (%)
SEMT, age, education	SCI	94.12 (0.76)	66.67 (0.85)	85.71 (0.56)	84.21 (0.86)
	aMCI(−)	95.12 (0.85)	45.45 (0.80)	71.43 (0.98)	86.67 (0.97)
	aMCI(+)	93.94 (0.79)	68.42 (0.68)	86.67 (0.90)	83.78 (0.83)
	AD	93.75 (0.86)	70.00 (0.70)	87.50 (0.80)	83.33 (0.96)
MMSE, age, education	SCI	91.62 (1.86)	53.84 (0.93)	85.98 (2.52)	67.45 (0.71)
	aMCI(−)	91.53 (1.46)	39.28 (1.04)	80.84 (2.61)	61.48 (0.83)
	aMCI(+)	91.53 (1.20)	44.64 (0.60)	82.19 (1.55)	65.36 (3.57)
	AD	91.12 (1.51)	54.61 (0.70)	86.09 (1.88)	66.59 (0.54)
SVLT, age, education	SCI	90.75 (1.17)	55.76 (0.65)	86.36 (1.74)	66.16 (3.48)
	aMCI(−)	91.41 (1.91)	32.14 (0.75)	78.98 (1.96)	56.99 (0.81)
	aMCI(+)	90.37 (1.46)	51.15 (0.87)	85.27 (2.29)	66.21 (0.67)
	AD	90.62 (1.37)	53.07 (0.74)	85.64 (1.91)	64.73 (3.74)

Radial basis function was used for the generation of the SVM classifier. The average and standard deviation (in parenthesis) were calculated from 20 repetition tests of 10-fold cross validation. For the SEMT based classifier, three different SEMT scores (free recall, recognition, and place matching scores) were used. For the SVLT based classifier, two SVLT scores (immediate and delayed scores) were used. AD: Alzheimer’s disease; aMCI(−): amyloid negative amnestic mild cognitive impairment; aMCI(+): amyloid positive amnestic mild cognitive impairment; NPV: negative predictive value; PPV: positive predictive value; SCI: subjective cognitive impairment; SVM: support vector machine.

## Discussion

We constructed a highly immersive test using HMD to allow participants to experience a 360° video and evaluated the performance of the participants who had been confirmed with amyloid PET in addition to neuropsychological tests and MRI. Consistent with previous studies, AD and amyloid positive aMCI participants demonstrated amyloid retention patterns in the bilateral frontal and temporoparietal cortices^14–16^. The major findings of this study are as follows. First, the SEMT scores highly correlated with conventional cognitive tests such as MMSE and SVLT scores, and hippocampal volume. Second, the logistic regression model indicated that SEMT scores can differentiate amyloid positive from amyloid negative groups with high accuracy. Third, SVM analysis yielded classifiers with high sensitivity and prediction accuracy for all four diagnostic groups: SCI, aMCI(−), aMCI(+), and AD. Taken together, our findings suggest that SEMT, which consists of a virtual reality social setting that can be experienced in everyday life, can be useful as a screening test.

Our first major finding was that all SEMT scores (free recall, place-matching, and total score), except recognition, highly correlated with existing standard tests (Fig. 2A). Interestingly, the SEMT total score correlated better with SVLT delayed recall (Fig. 2D) than SVLT immediate recall (Fig. 2C), suggesting that the SEMT has diagnostic value even though delayed recall components were not included. More specifically, whereas conventional memory tests such as the SVLT have several free recalls followed by delayed recall and recognition, the SEMT involves only free recall and recognition after watching a video. However, participants might have rehearsed the information while watching the 7-min video, which could have contributed to memory consolidation. The SEMT total score also showed a positive correlation with MMSE (Fig. 2B). This positive correlation of SEMT to conventional tests might be consistent with previous studies in which rendered image-based tests showed a correlation to standard tests, and these tests were feasible in elderly patients with aMCI and dementia^17^. Also, a recent study validated the reliability of HMD-based tests using the test-retest reliability of an HMD-based neuropsychological test^18^. Along with previous findings, the significant correlation of SEMT with conventional cognitive tests and hippocampal volume suggest the possibility of using VR-based neuropsychological tests as a screening test^19–23^.

One of the subtests of the SEMT was a facial recognition section where real and sham faces were randomly presented, and participants had to respond with either yes or no to the faces. Only the SEMT face recognition score did not differ among the three groups (p = 0.41), and the overall performances were similarly low. One reason might be that the resolution of the HMD device might not have been good enough for the participants to clearly recognize the faces. Therefore, future studies should incorporate a 360° camera and HMD device that can provide high resolution.

Our second major finding was that, based on logistic regression analysis, the SEMT had the ability to differentiate individuals with amyloid PET positivity from those with amyloid PET negativity (Fig. 3A). Although there is no significant logistic fitting model with conventional tests, additional ROC curve analysis indicated that the SEMT has comparable performance with conventional tests in terms of differentiating amyloid positive and negative individuals (Fig. 3B). This appealing result has two possible explanations based on findings from previous studies. One explanation is that, as mentioned above, the test could involve some delayed memory function since patients were tested after watching an approximately 7-minute-long video, although the SEMT was designed to be mostly a recall test. A previous study demonstrated that verbal delayed recall was significantly worse in the amyloid-positive group^3^. Also, based on longitudinal studies, poorer performance on delayed recall memory tasks predicted a higher rate of conversion to AD^24,25^. The other reason for the significant correlation between SEMT scores and amyloid PET positivity may be that the SEMT relies heavily on associative memory. It has been reported that associative memory is related to amyloid deposition^4,7^. In the present study, the SEMT tested associative memory by asking participants to verbally recall (SEMT free recall) or choose the right answer (SEMT place-matching) to questions about a particular actor (name, relationship, residing city, occupation, hobby, and gift). Therefore, delayed and associative memory components might have contributed to the association between SEMT scores and the presence of amyloid deposition from PET scans.

Based on logistic regression plots (Fig. 3A), it was apparent that most of the participants were correctly identified, and only two individuals were not predicted. Low amyloid deposition was observed in one participant who was actually amyloid-positive but predicted to be amyloid-negative based on the model. Low amyloid deposition might not affect the performance of the SEMT to a great extent. The other participant who was actually amyloid-negative but predicted to be amyloid-positive based on the model was found to have an EEG abnormality after participating in the study, which indicated that abnormal electrical potential in the brain can affect performance on the SEMT.

Our third major finding was that SVM analysis yielded classifiers with high sensitivity and prediction accuracy for all four diagnostic groups: amyloid negative SCI, aMCI(−), aMCI(+), and AD. Several studies have shown that SVM analysis using neuroimaging data (including structural MRI, functional MRI, DTI, and PET) provides a classification mechanism between normal controls and AD or aMCI patients^26,27^. In previous studies, SVM analysis using neuroimaging was able to predict which normal controls would convert to aMCI or AD^28,29^. However, our SVM analysis, which only incorporated a neuropsychological test, SEMT data, and demographic data, yielded classifiers with high prediction accuracy and sensitivity. This is probably because our SEMT was designed to reflect amyloid PET positivity.

In summary, we developed a test in which participants watched a 360° video to create a sense of immersion as if the participants were experiencing a real-life social event. Logistic regression analyses showed the association between SEMT data and amyloid PET positivity. Furthermore, SVM analysis of the SEMT data yielded sensitive classifiers for all four diagnostic groups. In addition, the SEMT might have several advantages over conventional cognitive tests in terms of dementia screening, although it was comparable to conventional tests in terms of differentiating amyloid positive and negative individuals. First, the administration time is relatively short. The MMSE is shorter than the SEMT, but is not sensitive enough to detect aMCI. In contrast, the SEMT assesses both verbal and visual memory in a relatively short time period by asking participants to remember the position of each actor (visuospatial memory) and the content of the conversation (episodic verbal memory). Second, since the SEMT is presented via an HMD, unlike conventional cognitive tests that expose people to different environments (e.g., room, light), the SEMT has the advantage of being less affected by environmental factors and the examiner. Furthermore, the SEMT does not require a trained psychometrician or neuropsychologist and, thus, can be easily administered in a community setting. Third, another advantage of the SEMT is that scores are not affected by age or years of education. This overcomes the limitation of existing screening tests such as the MMSE, which are influenced by educational level. This is probably due to the SEMT presentation of content experienced in everyday life and the lack of requirement for applying knowledge gained from education.

The study has several limitations. First, the number of participants was small since we only recruited participants confirmed by an amyloid PET scan, and the participants were restricted to individuals with more than 6 years of education who were 65 to 85 years old. Second, it was not possible to confirm whether the participants were attentive because the participants were wearing the HMD. In the future, the test can be improved by attaching an eye tracker to the HMD worn by the participants. Third, although prediction of amyloid positivity versus negativity was possible from the logistic analysis of the entire group, positive results were not obtained when the same analysis was performed on aMCI alone probably due to the small sample size. Therefore, future studies should recruit more participants. Furthermore, follow up studies using SEMT with various scenarios are needed to test and verify the results obtained in this study because only the birthday party scene was used. However, it is noteworthy that the SEMT developed in this study, which incorporated virtual reality to experience real-life events, showed potential to be used as a new memory screening test. The results of this study support the idea that the SEMT can provide a novel channel to perform diagnostic testing to identify individuals with cognitive impairment in its early stages.

## Methods

### Participants

We conducted the study in the Dementia Clinic at Samsung Medical Center from November 2016 to April 2017. All studies were performed in compliance with protocols approved by the institutional review board of Samsung Medical Center (IRB 2016-02-030) and written informed consent was obtained from all participants. We recruited the participants consecutively from the elderlies that satisfied the following conditions: (i) age range of 60 to 85 years old^14^, (ii) more than 6 years of education, (iii) normal visual acuity, (iv) absence of hearing impairment, (v) completion of a dementia work-up including neuropsychological tests and MRI, and (vi) amyloid PET scans. However, only participants who had received neuropsychological tests, MRI, and amyloid PET scans within one year from the SEMT test were included. For detailed neuropsychological tests, we assessed five cognitive domains: attention, language, visuospatial function, memory, and frontal/executive function using the Seoul Neuropsychological Screening Battery-Dementia version as well as MMSE^30^ and SVLT^31^. Experienced neurologists evaluated the participants based on their clinical symptoms and reviews of medical/medication history, neuropsychological test results, neuroimaging data (MRI, PET), and laboratory tests, and then classified the participants into three diagnostic groups: SCI, aMCI and AD. In brief, the diagnosis of AD was based on the criteria proposed by the National Institute of Neurological and Communicative Disorders and Stroke and the Alzheimer’s Disease and Related Disorders Association (NINCDS-ADRDA)^32^. In order to have comparable groups, we selected early-stage of AD patients showing mild dementia defined by MMSE scores ≥20 and clinical dementia rating (CDR) ≤1). For the AD group, only patients showing amyloid positivity were included. The diagnosis of aMCI was based on the criteria proposed by Peterson and colleagues^33^. The aMCI group was then divided into amyloid negative [aMCI(−)] and positive [aMCI(+)] groups according to amyloid PET positivity. SCI was the group of individuals who self-reported persistent decline in cognitive/memory capacity but were not impaired on neuropsychological tests^34^. The absence of presymptomatic or prodromal AD was also confirmed by negative amyloid PET scans in 11 of 13 SCI individuals. In all the three groups, we excluded participants with other structural lesions such as territorial infarction, intracranial haemorrhage, brain tumour, hydrocephalus, or severe white matter hyperintensities (WMH) observed on brain MR images. Severe WMH on MRI was defined as a cap or a band ≥10 mm as well as a deep white matter lesion ≥25 mm as modified from the Fazekas ischemia criteria. None of the participants had a family history suggestive of an autosomal dominant disease. Based on the aforementioned criteria, a total of 52 participants were recruited and were divided into the following groups: SCI (n = 13), aMCI(−) (n = 9), aMCI(+) (n = 16), and AD (n = 14). Figure 1 shows the voxel-based statistical parametric mapping of PET amyloid ligand [C-11] uptake comparing between groups. Table 1 summarizes the demographic and clinical characteristics.

### MR imaging and [18 F] florbetaben PET imaging analysis

Details on image acquisition and analyses are described in the Supplementary Methods.

### Experiments

**Figure 5:**
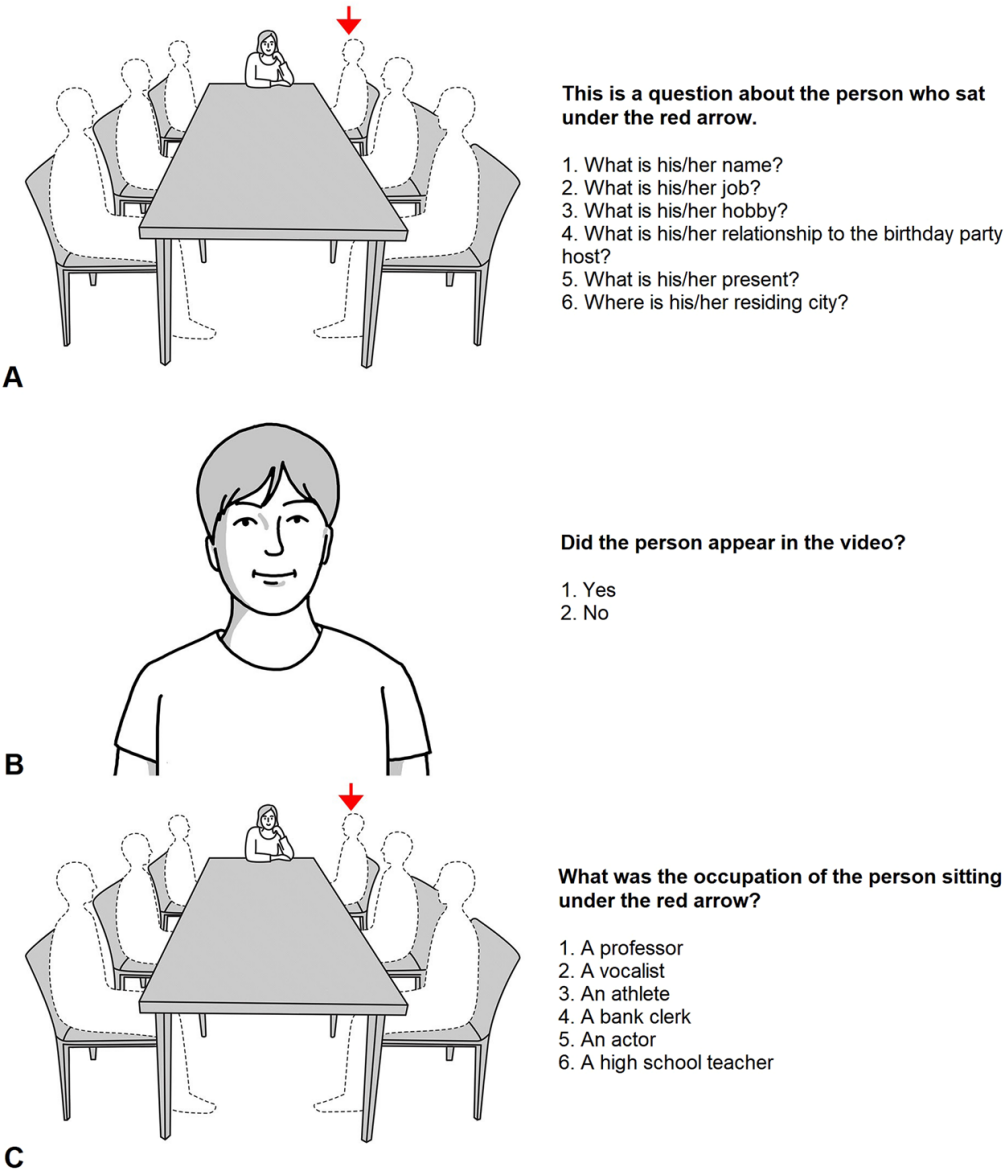
Participants were instructed to respond after viewing a photograph of the room where the birthday party took place in the video. In the actual experiment, a photograph was shown, which has been replaced with an illustration here. The host is seated in the centre, and the guests are illustrated as silhouettes. (**A**) Free recall, (**B**) recognition, and (**C**) place-matching tests were conducted using the photographs. The waiting period varied depending on the subject’s condition, but there was approximately 1 min between the end of the video and start of the questionnaire. Normally, it took 20–30 min for participants to finish the questionnaire. All participants responded to all 102 questions. (permission is granted to Macmillan Publishers Ltd, part of Springer Nature).

Before the experiment, all participants completed tests for visual acuity and hearing. Participants with visual acuity below 0.3 or impaired hearing were excluded from this study. Before watching the video, participants were instructed to remember the dialogue between the actors as much as possible. After watching the video, the memory test was performed using a laptop computer (15 inch, 1920 × 1080), which was placed approximately 30 cm in front of the participants. The memory test included free recall, facial recognition, and place-matching tests (Fig. 5). The scoring system is described in Supplementary Table S6. The participants responded by pressing a button on a Bluetooth keyboard (82 × 124 × 1  mm).

#### Movie for the assessment of social event memory

A 7.5-min-long movie was developed for the assessment of social event memory. Detailed specifications of the 360° camera and HMD used for shooting and testing are described in the Supplementary Methods. The video showed a scene where the host was having a birthday party in a room that a professional party planner had created. As illustrated in Fig. 5A, the host (birthday person) sat in the middle of a rectangular table with three guests sitting symmetrically on each the left and right of the host. The scene begins with everybody singing the birthday song, followed by the host’s brief greeting and then each guest taking turns introducing themselves by saying the following: (1) name, (2) relationship to the host, (3) current city of residence, (4) vocation, and (5) hobby. At the end of each speaker’s introduction, a birthday gift is presented to the host. Information provided by each of the guests (relationship to the host, vocation, hobby, and gift) was mentioned twice: once from the speaker himself and the second from another guest who repeated the information provided by the speaker. The video ends with one guest recalling the name and city of residence of another guest. As a result, all participants were able to hear the information on each of the respective guests twice.

Theatre actors with over 10 years of experience in the acting profession played the host and guests. The host or birthday person was not the target of the test. The six guests consisted of three females and three males. There were three young actors (26.0 ± 2.3 yrs., female:male = 2:1) and three middle aged actors (50.3±4.9 yrs., female:male = 2:1).

#### Free recall test

In the free recall test, a photograph of the room where the birthday party took place in the video was shown. An illustration of the photograph is shown in Fig. 5A. The illustration shows the seating arrangement of the host and guests who are drawn as silhouettes. A guest was randomly chosen (marked with a red arrowhead in Fig. 5A), and the participants had to recall the information provided by the respective guest (name, relationship to host, city of residence, occupation, hobby, and gift presented to the host) within 2 minutes. When participants finished recalling as much information as possible or could not remember further, they were then instructed to recall the information of another guest, indicated by a red arrow in Fig. 5A. Participants were not allowed to change their responses after proceeding to the next question (Fig. 5A).

### Facial recognition test

In the facial recognition test, 18 photographs of different faces were prepared, 6 of which were faces of the actors who played the guests in the video clip, and 12 were sham faces of people who did not appear in the video. Participants were presented with the 18 photographs one at a time on a laptop screen and were instructed to answer whether each face appeared in the video by pressing the O or X buttons. The next photograph was presented to the participant after the button was pressed. If participants did not respond within 20 seconds after seeing a photograph, the response was considered incorrect, and the next photograph was presented. In order to eliminate the effects of non-facial elements such as clothes and accessories in facial recognition, all 6 actors and 12 people were photographed wearing an identical white shirt without any accessories. Also, there were no significant differences in terms of gender ratio and age between the 6 real and 12 sham faces (Fig. 5B).

#### Place-matching test

In the place-matching test that was designed to assess association memory, participants were shown the illustration in Fig. 5C, where one of the six guests was marked with a red arrowhead. Participants had to choose the correct name of the chosen guest among six choices, consisting of three sham names and three additional names of the guests that appeared in the video (out of these three, one was the correct name). Therefore, there were a total of 36 names (6 places $\times$ 6 choices): 18 sham and 18 real names. Each real name was presented three times, and each sham name was presented once or twice. The names were presented randomly, but in the same order for all participants. Participants were asked to respond to the questions by pressing one of the keyboard buttons (indicating one to six) within 20 seconds. If an answer was not given within 20 seconds, the response was considered incorrect (Fig. 5C).

The same procedure was performed for the other categories (relationship, residing city, occupation, hobby, gift, face, and accessories that the guests were wearing such as a cap). Therefore, participants received a total of 48 questions (6 questions per category $\times$ 8 categories). All questions were multiple choice: 5 of the 8 categories (name, relationship, residing city, occupation, and hobby) were presented in written words, and the remaining 3 categories (gifts, faces, and accessories) were presented with photographs. The order of the categories was same order for all participants.

### Statistical analysis

The statistical analysis was performed using non-parametric tests with a significance level of 0.05. For the same sample size, the Kruskal-Wallis test was used, otherwise the Mann–Whitney U test was used. The results are presented as mean ± standard deviation. A two-sample t-test was used for continuous variables, and a chi-square test was used for categorical variables. Pearson’s correlations were calculated to test whether test scores and age/education were correlated. SVM is a well-known classification technique in constructing data-driven classifiers. SVM performs classification by finding a hyperplane that maximally separates the data into two categories. SVM is usually applied with a kernel function to transform input data into hyperspace. Classification methods are widely used in many applications of medical diagnosis. Medical diagnosis, referred as allocation in the older statistic, is an archetypal classification problem^35^. These classification tools are supervised learning methods where the algorithm learns from a training set and establishes a prediction rule to classify new samples using statistical approaches for class prediction. Details on logistic regression analysis and SVM analyses are described in the Supplementary Methods.

## Electronic supplementary material

Supplementary Info
