# Supplementary material for: Social Event Memory Test (SEMT): A Video-based Memory Test for Predicting Amyloid Positivity for Alzheimer’s Disease
Source: Sci Rep. 2018 Jul 10;8:10421. doi: 10.1038/s41598-018-28768-1 (PMC6039498; doi:10.1038/s41598-018-28768-1)
Supplement: Supplementary file 1 — Supplementary Info [file 41598_2018_28768_MOESM1_ESM.docx]

**Social Event Memory Test (SEMT): A Video-based Memory Test for Predicting Amyloid Positivity for Alzheimer’s Disease**

Ko Woon Kim^1,2^, Jong Doo Choi^1^, Hyejoo Lee^3^, Na Kyung Lee^4^, Seongbeom Park^1^, Juhee Chin^1^, Byung Hwa Lee^1^, Jiwon Shin^5^, Yeshin Kim^1^, Hyemin Jang^1^, Jee Hyun Choi^3^*, Duk L. Na^1,6,7^*

^1^ Department of Neurology, Samsung Medical Center, Sungkyunkwan University School of Medicine, Seoul, Korea

^2^ Department of Neurology, Chonbuk National University Medical School & Hospital, Jeonju, Korea

^3^ Convergence Research Center for Diagnosis, Treatment, and Care System of Dementia, Center for Neuroscience, Korea Institute of Science and Technology, Seoul, Republic of Korea

^4^ Department of Health Sciences and Technology, SAIHST, Sungkyunkwan University, Seoul, Republic of Korea

^5^Sungkyunkwan University School of Medicine, Seoul, Korea

^6^ Neuroscience Center, Samsung Medical Center, Seoul, Korea

^7^ Department of Clinical Research Design & Evaluation, SAIHST, Sungkyunkwan University, Seoul, Korea

***Corresponding authors**

**Duk L. Na, MD, PhD**

Department of Neurology, Samsung Medical Center, Sungkyunkwan University School of Medicine, 81 Irwon-Ro, Gangnam-gu, Seoul, 06351, South Korea

Tel.: +82-2-3410-3591/3599

Fax: +82-2-3410-0052

E-mail: dukna@skku.edu

**Jee Hyun Choi**

Convergence Research Center for Diagnosis, Treatment, and Care System of Dementia, Center for Neuroscience, Korea Institute of Science and Technology, Hwarangno 14-gil 5, Seongbuk-gu, Seoul 136-791, South Korea

Tel.: +82-2-958-6952

Fax: +82-2-958-6937

E-mail: jeechoi@kist.re.kr

**Supplementary Methods**

***MR imaging and [18F] florbetaben PET imaging analysis***

All patients underwent MRI using the same 3.0 T MRI scanner (Philips 3.0T Achieva, The Netherlands), and [18F] florbetaben PET scans were obtained using a Discovery STe PET/CT scanner (GE Medical Systems, Milwaukee, WI, USA) at Samsung Medical Center. The quantification methods for florbetaben retention are described below in “Quantification of florbetaben retention. To compare the distribution of florbetaben retention among the four groups (SCI, amyloid negative aMCI, amyloid positive aMCI, and AD), a voxel-based statistical analysis was performed using SPM8 and Matlab 6.5 for Windows (Math Works, Natick, MA, USA). A detailed explanation of the method is described in our previous study.

**1) Quantification of florbetaben retention**

Florbetaben PET images were co-registered to individual MRIs, which were normalized to a T1-weighted MRI template. Using these parameters, MRI co-registered florbetaben PET images were normalized to the MRI template. The quantitative regional values of florbetaben retention on the spatially normalized florbetaben images were obtained using an automated volume of interest (VOI) analysis with the automated anatomical labeling (AAL) atlas. Data processing was performed using the Statistical Parametric Mapping program, Version 8 (SPM8) within the Matlab 6.5 software package (Mathworks, Natick, MA, USA). To measure florbetaben retention, we used the standardized uptake value ratios (SUVRs) with the cerebellum as a reference region where there were minimal group differences. We selected 28 cortical VOIs from each hemisphere using the AAL atlas. The cerebral cortical VOIs chosen for this study consisted of the bilateral frontal areas (the superior and middle frontal gyri, the medial part of the superior frontal gyrus, the opercular part of the inferior frontal gyrus, the triangular part of the inferior frontal gyrus, the supplementary motor area, the orbital part of the superior, middle, and inferior orbital frontal gyri, the rectus, and the olfactory cortex), posterior cingulate gyri, parietal areas (superior and inferior parietal areas, supramarginal and angular gyri, and precuneus), lateral temporal areas (superior, middle, and inferior temporal gyri and Heschl’s gyri), and occipital areas (superior, middle, and inferior occipital gyri, cuneus, calcarine fissure, and lingual and fusiform gyri). Regional cerebral cortical SUVRs were calculated by dividing each cortical VOI’s SUV by the mean SUV of the cerebellar cortex value (cerebellum crus1 and crus2). A global florbetaben uptake ratio was calculated from the volume-weighted average SUVR of 28 cerebral cortical VOIs from each hemisphere. Pre-defined regions of interest (ROIs) included (separately for the left and right hemispheres): the frontal cortex (anterior to precentral gyrus), precuneus, posterior cingulate cortex, lateral parietal cortex, lateral temporal cortex, occipital cortex, and striatum. As a measure of global amyloid burden, we calculated a florbetaben index, representing the subject’s mean distribution volume ratio in the frontal, parietal, lateral temporal, and precuneus-posterior cingulate cortices (PC-PCC) and the striatum ^1^.

**2) Voxel-wise group comparison of florbetaben PET images**

SPM analysis was performed without global count normalization, since the [18F] florbetaben PET images had been changed to the uptake ratio (SUVR) parametric image using the cerebellar ROI uptake value. Statistical comparisons between groups were performed on a voxel-by-voxel basis using t-statistics. To compare between groups [aMCI (-) vs. SCI, aMCI (+) vs. SCI, aMCI (+) vs. aMCI (-), AD vs. SCI, AD vs. aMCI (-), AD vs. aMCI (+)], we investigated the increased florbetaben retention areas of the brain at a height threshold of FWE corrected p value *<* 0.05 and at an extent threshold of 150 voxels. The MNI coordinates of the local maximum of each cluster were converted into Talairach coordinates.

3) **Correlation analysis of SEMT score with hippocampal volume**

We obtained the right, left, and total hippocampal volume of each participants through an automated hippocampal segmentation method based on a graph-cuts algorithm combined with atlas-based segmentation and morphological opening.^2^ After that, Pearson’s correlations were calculated to test whether hippocampal volume and SEMT scores were correlated. Also, Pearson’s correlations were calculated to test whether hippocampal volume and other conventional cognitive test scores (MMSE and SVLT) were correlated.

***Detailed specifications of the 360° camera and HMD***

To improve the sense of immersion as well as to control the environment, the movie was filmed with a 360° camera (Gear 360, 3840×1920, 30 fps, Samsung Inc., South Korea) and presented to participants through a head mounted display (HMD, Gear VR, Samsung Inc., South Korea) operated by a smartphone (534 pixels per inch, Galaxy S7, Samsung Inc., South Korea). The video showed a scene where the host was having a birthday party in a room that a professional party planner had created. As illustrated in Figure 2, the host (birthday person) sat in the middle of a rectangular table with three guests sitting symmetrically on each the left and right of the host.

***Statistical analyses***

**1) Logistic regression analysis to investigate whether SEMT score can differentiate amyloid positive and negative groups**

In order to build a prediction model for amyloid positivity/negativity based on SEMT score, logistic regression analysis was performed.^4^ The dependent variable was binary, *i.e.*, 1 or 0 for amyloid positivity or negativity, respectively. As independent variables, SEMT recognition/free recall/place matching/total scores and age/education were chosen based on correlation analysis. In the logistic regression model, the probability of the outcome is predicted, and the range of outcomes is bound between 0 and 1. A logic function was used, and the logit of the outcome variable was presented in linear combinations: *x_i_* or (*x_i_ – const*) × (*x_j_ – const*), where {*x_i_*} is the independent variable group, and *i* and *j* are variable indexes. For a given data set, all-possible-regressions were built by successively adding or removing variables.

The performances of the logistic regression models were compared by Akaike information criterion (AIC) where the predictive power was evaluated using R-square statistics for the distance between fitted and true values.^5^ The best fitting model (saturated model) was found by minimizing AIC with a high R-squared. In choosing a model, it is important not only to satisfy goodness-of-fit to the known dataset, but also to generalize the unknown data set. Therefore, the comprehensive approach reported by Miller and colleagues was followed to validate the models.^6^ Briefly, the likelihood ratio test (LRT) was performed to compare the goodness-of-fit of each model to the saturated model, and then the significance of each coefficient was tested using the bias-variance decomposition method. Through this approach, a simpler model than the saturated model can be chosen.

In this study, model with all main effects included was considered as a preliminary model. The preliminary model fit the data poorly, with an Akaike information criterion (AIC) of 36.88 and R-squared value of 0.69. Along with the main effect, interactions and quadratic effects were also included, and their regression fits were compared. The significantly correlated variables obtained from the correlation matrix were considered as the interaction terms. The optimal regression model with an AIC of 25.95 and high R-squared of 0.78 was selected.

**2) Classification of the four groups using the Support Vector Machine SEMT classifiers**

To develop a statistical classifier to classify the four different groups (*i.e.*, SCI, MCI (–), MCI (+), and AD), the support vector machine (SVM) was used. We implemented the SVM classifier using the R-package Lib SVM.^7^ In this study, linear and RBF kernels were used. Age, education, and SEMT scores served as inputs to the SVM, and the subject type was the output of the SVM.

The optimal SVM model was obtained by grid-search, which is the commonly used optimization method. The grid search method finds the various pairs of (C, γ), where C is the penalty parameter, and γ is the kernel parameter.^7^ The optimal model is selected as that with the most accurate C and γ. In order to prevent the over-fitting problem due to the small number of observations, we have conducted twenty repetitions of the 10-fold cross-validation technique. The K-fold cross-validation procedure is known as a model validation technique which can prevent the overfitting problem (Lin et al., 2003). In K-fold cross-validation, the original sample is randomly partitioned into K subsamples. Of the K subsamples, a single subsample is retained as the validation data set for testing the model, and the remaining K – 1 subsamples are used as training data. Ten-fold repetition was chosen because accuracy has been reported to be nearly saturated at K=10. ^9^ For 10-fold cross-validation, 90% of the data is retained as a training set and the remaining 10% of the data is put into the validation data set. The cross-validation technique is then repeated 10 times (the folds). The statistical results of twenty repetitions of 10-fold cross-validation were averaged and reported.

To measure the classification performance, sensitivity, specificity, positive predictive value (PPV), and negative predictive value (NPV) were calculated separately and then averaged. The efficacy of these models on these data was evaluated by overall accuracy, sensitivity, specificity, PPV and NPV. Briefly reviewed, overall accuracy was obtained by the total number of correct predictions divided by the total number of predictions. Sensitivity measured the proportion of positive predictions among true positives and specificity measured the proportion of negative predictions among true negatives. PPV is the proportion of true positives among the positive predictions, and NPV is the proportion of true negatives among the negative predictions. In our study, cases with a disease are regarded as people in the positive class, and the control group is regarded as the negative class.

**Supplementary Tables**

**Supplementary Table S1. Demographic and clinical characteristics in the aMCI subgroup**

|  | | **Mean ± SD** | | | **P** | |
| --- | --- | --- | --- | --- | --- | --- |
|  |  | **aMCI(-)** | **aMCI(+)** | |  |  |
| **N** | 9 | | | 16 | |  |
| **Age**, years | 75.44 ± 4.53 | | | 75.50 ± 5.16 | | 0.9788 |
| **Education**, years | 12.11 ± 5.40 | | | 13.38 ± 4.29 | | 0.4667 |
| **Gender** |  | | |  | |  |
| Female:Male | 5:4 | | | 9:7 | | 1 |
| **APOE4 carrier**  **(%)** | 1/9(11.1%) | | | 10/16(62.5%) | | 0.0330 |
| **MMSE** | 26.56 ± 2.96 | | | 25.25 ± 2.77 | | 0.1987 |
| **Domain score**  **(max score)** |  | | |  | |  |
| Attention (17) | 8.67 ± 2.06 | | | 9.56 ± 2.71 | | 0.4211 |
| Language (27) | 21.33 ± 4.66 | | | 21.19 ± 3.58 | | 0.4586 |
| Visuospatial (36) | 27.06 ± 8.79 | | | 31.06 ± 3.23 | | 0.5314 |
| Memory (150) | 57.28 ± 12.65 | | | 41.22 ± 11.53 | | 0.0037 |
| Frontal/Executive (70) | 41.33 ± 14.99 | | | 46.44 ± 11.31 | | 0.2452 |
| Total (300) | 155.67±32.40 | | | 149.47±24.70 | | 0.6103 |
| **SVLT**  **(max score)** |  | | |  | |  |
| Immediate (36) | 16.67 ± 3.39 | | | 15.27 ± 4.17 | | 0.4039 |
| Delayed (12) | 3.56 ± 1.88 | | | 1.13 ± 1.25 | | 0.0026 |
| **SEMT**  **(max score)** |  | | |  | |  |
| Free recall (36) | 8.00 ± 5.70 | | | 1.75 ± 2.08 | | 0.0034 |
| Recognition (18) | 11.56 ± 1.81 | | | 10.81 ± 1.52 | | 0.2835 |
| Place-matching (48) | 15.67 ± 8.60 | | | 7.44 ± 4.56 | | 0.0045 |
| Total (102) | 35.22 ± 14.15 | | | 20.00 ± 6.59 | | 0.0012 |

**aMCI (-): amyloid negative amnestic mild cognitive impairment; aMCI (+): amyloid positive amnestic mild cognitive impairment; MMSE: Mini Mental State Examination; SEMT: social event memory test; SVLT: Seoul Verbal Learning Test**

**Supplementary Table S2.** Comparison between amyloid (-) and (+) subjects in the subgroup with normal MMSE

|  | Amyloid negative  (N = 14) | Amyloid positive  (N = 3) | P |
| --- | --- | --- | --- |
| Age | 72.79/4.35 | 79.67/3.51 | 0.037 |
| Education | 15/2.29 | 15/1.73 | 0.825 |
| MMSE (30) | 29.21/0.699 | 28.67/0.577 | 0.211 |
| **SEMT total (102)** | **56.43/13.01** | **19.00/8.72** | **0.008** |
| **SEMT free recall (36)** | **16.57/5.69** | **3.00/2.65** | **0.014** |
| SEMT recognition (18) | 11.57/2.24 | 10.00/1.00 | 0.200 |
| **SEMT place-matching (48)** | **28.29/8.64** | **6.00/5.20** | **0.008** |

Statistical significance test was done by Mann-Whitney U-test.

**MMSE: Mini Mental State Examination; SEMT: social event memory test**

**Supplementary Table S3.** Results of correlation analysis between Apo E genotypes and SEMT scores in the aMCI group

|  | Apo E genotype | |
| --- | --- | --- |
|  | r | P |
| SEMT total | - 0.373 | 0.073 |
| SEMT free recall | -0.243 | 0.234 |
| SEMT recognition | -0.165 | 0.441 |
| **SEMT place-matching** | **-0.422*** | **0.040** |

* *p* <.05 for Pearson correlation coefficient.

**SEMT: social event memory test**

**Supplementary Table S4.** Pairwise correlation

| **Variable** | **by Variable** | **Correlation** | **Count** | **p** |
| --- | --- | --- | --- | --- |
| SEMT free recall | SEMT total | 0.9577 | 52 | <0.0001 |
| SEMT place-matching | SEMT total | 0.9833 | 52 | <0.0001 |
| SEMT place-matching | SEMT free recall | 0.9090 | 52 | <0.0001 |
| SVLT delayed | SEMT total | 0.7943 | 50 | <0.0001 |
| SVLT delayed | SEMT free recall | 0.8052 | 50 | <0.0001 |
| SVLT delayed | SEMT place-matching | 0.7610 | 50 | <0.0001 |
| SVLT immediate | SEMT total | 0.7131 | 50 | <0.0001 |
| SVLT immediate | SEMT free recall | 0.7298 | 50 | <0.0001 |
| SVLT immediate | SEMT place-matching | 0.6824 | 50 | <0.0001 |
| SVLT immediate | SVLT delayed | 0.8087 | 50 | <0.0001 |
| MMSE | SEMT total | 0.6862 | 52 | <0.0001 |
| MMSE | SEMT free recall | 0.6754 | 52 | <0.0001 |
| MMSE | SEMT place-matching | 0.6788 | 52 | <0.0001 |
| MMSE | SVLT delayed | 0.6970 | 50 | <0.0001 |
| MMSE | SVLT immediate | 0.6563 | 50 | <0.0001 |
| Age | SEMT total | -0.1483 | 52 | 0.2942 |
| Age | SEMT free recall | -0.1160 | 52 | 0.4129 |
| Age | SEMT place-matching | -0.1488 | 52 | 0.2924 |
| Age | SVLT delayed | -0.1595 | 50 | 0.2687 |
| Age | SVLT immediate | -0.1726 | 50 | 0.2308 |
| Age | MMSE | -0.1097 | 52 | 0.4390 |
| Education | SEMT total | 0.0975 | 52 | 0.4917 |
| Education | SEMT free recall | 0.0641 | 52 | 0.6516 |
| Education | SEMT place-matching | 0.1133 | 52 | 0.4238 |
| Education | SVLT delayed | 0.1161 | 50 | 0.4218 |
| Education | SVLT immediate | 0.0334 | 50 | 0.8180 |
| Education | MMSE | 0.3247 | 52 | 0.0188 |
| Education | Age | 0.2874 | 52 | 0.0388 |

**Supplementary Table S4** shows the correlation of SEMT score with SVLT, MMSE, age, and education. The correlations between the SEMT place-matching, SEMT total, and SEMT free recall scores are all above 0.9 (p<0.0001). Significant correlations between SEMT and SVLT scores were also observed (p<0.0001). A high MMSE score is significantly associated with high SEMT scores (p<0.0001). The significance level have been adjusted $\alpha=\frac{\alpha}{8}=0.00625$ using a Bonferroni adjustment.

**SEMT: social event memory test; SVLT: Seoul Verbal Learning Test**

**Supplementary Table S5.** Results obtained from logistic regression analysis of amyloid PET positivity or negativity according to SEMT free recall and SEMT total scores

| **Output for fitting model** | | | |  |  |
| --- | --- | --- | --- | --- | --- |
| **Model** | **DF** | **-Log Likelihood** | | **Chi-square** | **P >Chi** |
| Difference | 4 | 26.36 | | 52.71 | <0.0001 |
| Full |  | 7.29 | |  |  |
| Reduced |  | 33.65 | |  |  |
| R-squared (U) | 0.78 |  | |  |  |
| AIC | 25.95 |  | |  |  |
| **Lack of Fit** |  |  | |  |  |
| **Model** | **DF** | **-Log Likelihood** | | **Chi-square** | **P >Chi** |
| Full | 36 | 7.29 | | 14.59 | 0.9994 |
| Saturated | 40 | 0.001 | |  |  |
| Difference | 4 | 7.29 | |  |  |
| **Likelihood Ratio Statistics** | | | | | |
| **Term** | | **DF** | **L-R Chi-square** | | **P >Chi** |
| SEMT total | | 1 | 24.70 | | <0.0001 |
| SEMT total*SEMT total | | 1 | 8.82 | | 0.0030 |
| SEMT total*SEMT free recall | | 1 | 9.38 | | 0.0022 |
| SEMT free recall*SEMT free recall | | 1 | 5.21 | | 0.0225 |

**Supplementary Table** **2** shows the results obtained from logistic regression analysis. Negative log likelihood indicates the variation of fitting measures. Negative log likelihood of the full model is obtained after fitting the model, and a reduced model is obtained when the model does not have any effects. The likelihood ratio test is designed to assess the significance of coefficients using likelihood ratio chi square statistics. The significant coefficients of SEMT total indicated its high contribution to the model, and significant associations were noted between SEMT total scores and PET outcome (p<0.0001). The quadratic effect of the SEMT total score showed its high contribution to predicting amyloid PET outcome (p=0.003). PET results can also be predicted by the interaction effect between SEMT total and SEMT free recall scores (p=0.0022). The quadratic term of SEMT free recall showed significant associations to PET scan results (p=0.225).

**Supplementary Table S6.** SEMT scoring system

| **Subitems** | **SEMT**  **free recall** | **SEMT recognition** | **SEMT**  **place-matching** | **SEMT**  **total** |
| --- | --- | --- | --- | --- |
| **Number of questions** | 6 questions for each seating of the 6 guests | 18 O/X questions | 8 questions for each seating of the 6 guests | Free recall + recognition + place-matching |
| **Score** | 36 | 18 | 48 | 102 |

**SEMT: social event memory test**

**References**

1 Noh, Y. *et al.* The Role of Cerebrovascular Disease in Amyloid Deposition. *J Alzheimers Dis* **54**, 1015-1026, doi:10.3233/JAD-150832 (2016).

2 Kwak, K. *et al.* Fully-automated approach to hippocampus segmentation using a graph-cuts algorithm combined with atlas-based segmentation and morphological opening. *Magn Reson Imaging* **31**, 1190-1196, doi:10.1016/j.mri.2013.04.008 (2013).

3 Sabri, O. *et al.* Florbetaben PET imaging to detect amyloid beta plaques in Alzheimer's disease: phase 3 study. *Alzheimers Dement* **11**, 964-974, doi:10.1016/j.jalz.2015.02.004 (2015).

1. Cox, D. R. The Regression analysis of binary sequences, *J Roy Stst Soc B Met* **20 (2)**, 215-242 (1958).
2. Agresti, A. An Introduction to categorical data analysis. 2nd ed. (Wiley Press, 2006).
3. Miller, M. E, Hui, S. L &Tierney, W. M. Validation techniques for logistic regression models, *Stat Med*. 10, 1213–1226 (1991).
4. Chang, C. C., & Lin, C. J. LIBSVM: a library for support vector machines. *Acm T Intel Syst Tec*, **2^3^**, 27. (2011)
5. Cortes, C & Vapnik, V. N. Support vector networks, *Mach. Learn*. **20**, 273-297 (1995).
6. Kohavi, R. A study of cross-validation and bootstrap for accuracy estimation and model selection. In C. S. Mellish (Ed.). *Proceedings of the 14th International Joint Conference on Artificial Intelligence,* **6(4)**, 1137–1143 (1995).
